# Sturge-Weber syndrome coexisting with polydactyly: a case report

**DOI:** 10.1186/s12886-020-01761-x

**Published:** 2021-01-06

**Authors:** Hongxi Wang, Nana Dong, Li Tan, Chukai Huang

**Affiliations:** Joint Shantou International Eye Center of Shantou University and the Chinese University of Hong Kong, North Dongxia Road, Shantou, 515041 Guangdong China

**Keywords:** Sturge-Weber syndrome, Polydactyly, Port-wine stains, Comorbidity, Case report

## Abstract

**Background:**

Sturge-Weber syndrome (SWS) is a sporadic congenital disorder, characterized by unilateral facial nevus flammeus associated with ipsilateral glaucoma, choroidal angioma and leptomeningeal hemangiomas. SWS can comorbid with other disorders in some patients, however, there has been no prior described case of SWS and polydactyly occurring in the same patient.

**Case presentation:**

A 15-year-old girl with diagnosis of SWS presented to our hospital. She had bilateral glaucoma and extensive port-wine stains distributing in bilateral faces, left neck and left upper limb. Meanwhile, the patient was noted to demonstrate the superfluous digit attaching on the left thumb and was diagnosed as polydactyly. Trabeculectomy, with intraoperative application of mitomycin C and postoperative subconjunctival injections of 5-fluorouracil, was successful in controlling the intraocular pressure in both eyes.

**Conclusions:**

We report a case with bilateral SWS coexisting with unilateral polydactyly, which, to our knowledge, has not been recognized previously and adds further evidence to the existing literature. In view of the rare concurrence of SWS and polydactyly, the etiology is unclear and further investigation is required to explore the underlying pathogenesis.

## Background

Sturge-Weber syndrome (SWS) is a neuro-oculocutaneous disorder, characterized by unilateral facial nevus flammeus associated with ipsilateral vascular malformation involving the eye and brain. SWS is a congenital and sporadic disease with an estimated incidence of 1 in 20,000 to 50,000 live births without gender or ethnic preferences [1]. The facial nevus flammeus of SWS is typically flat to moderately thick port-wine stains (PWS) or birthmarks following the distribution of the trigeminal nerve [2]. Ocular involvement can be found in up to 70% of cases [1], including glaucoma, choroidal hemangioma and telangiectasia of conjunctiva or sclera. Besides, leptomeningeal hemangiomas can be present and causes the atrophy of the cortical parenchyma of the brain, seizures, migraine, and cognitive impairment [3]. In a few patients, SWS can co-exist with other co-morbidities involving endocrinal or skeletomuscular system [4–9]. However, polydactyly has not been reported in patients with SWS. Here we report a case of bilateral glaucoma associated with extensive nevus flammeus and additionally, unilateral polydactyly. To our knowledge, this is the first case with co-existence of SWS and polydactyly.

## Case presentation

A 15-year-old girl was admitted to our hospital with chief complaint of progressive visual loss in both eyes for 3 years. No ocular redness, pain, photophobia or tearing was noticed. She denied the experience of seizures, migraines or behavioral disorders. She was the full-term product of a normal pregnancy and delivery, remarkable only for her extensive port-wine birthmarks and the extra finger on the left hand. Her growth and development were normal. Her parents, sister and two brothers were healthy without the related signs.

**Fig. 1:**
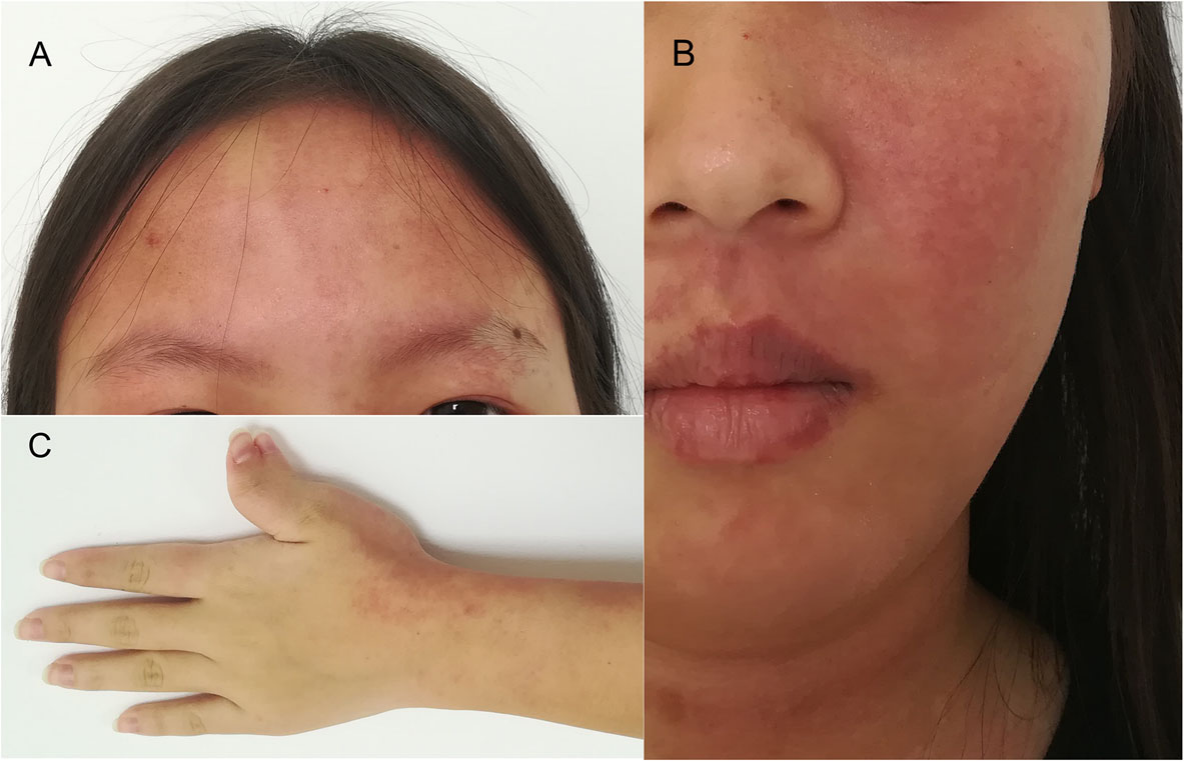
Cutaneous nevus flammeus and polydactyly. Cutaneous nevus flammeus distributing in **a** the forehead bilaterally, **b** the left cheek, jaw, neck, as well as **c** the left forearm. Superfluous digit on the radial side of the left thumb (biphalangeal thumb), with the distal digits deviating radially (**c**)

**Fig. 2:**
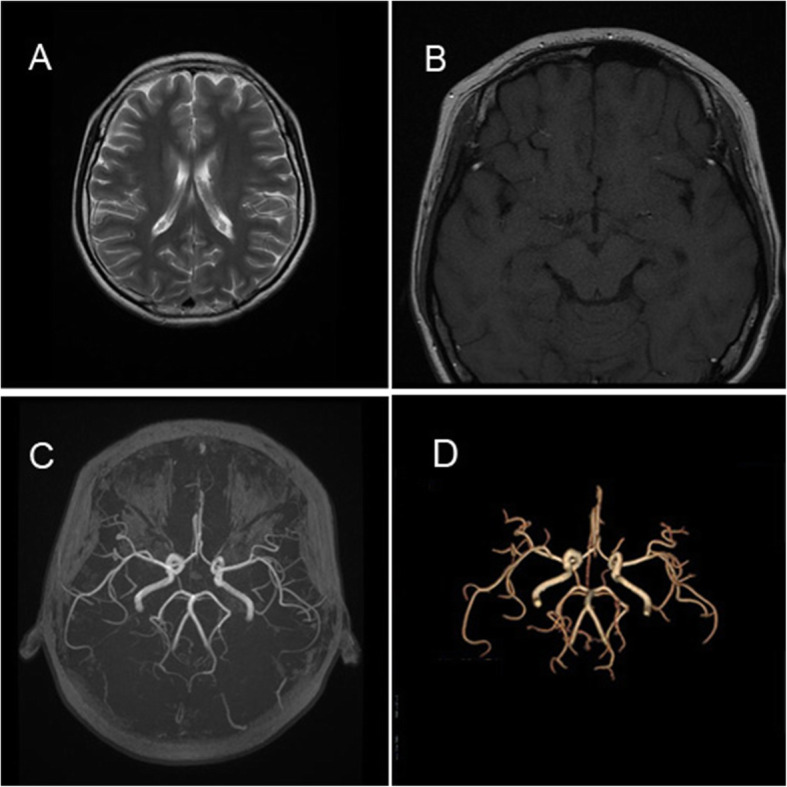
Neuroimaging. An axial cranial fast spin-echo T2-weighted (**a**) and T1-weighted (**b**) magnetic resonance imaging revealed no cerebral calcification or atrophy. Three-dimensional time-of-flight (**c**) and volume rendering (**d**) for magnetic resonance angiography demonstrates no intracranial leptomeningeal angiomatosis

General physical examinations revealed an otherwise healthy female with nevus flammeus affecting the bilateral forehead (Fig. 1a) and extending to the left cheek, jaw, neck and forearm (Fig. 1b and c). Moreover, the lips and the palatine mucosa were also involved. Superfluous digit was found attaching on the radial side of left thumb, and distal digits of the biphalangeal thumb deviated radially (Fig. 1c). No remarkable abnormal neurological sign was found. The neuro-imaging demonstrated no leptomeningeal vascular malformations or cerebral atrophy (Fig. 2).

**Fig. 3:**
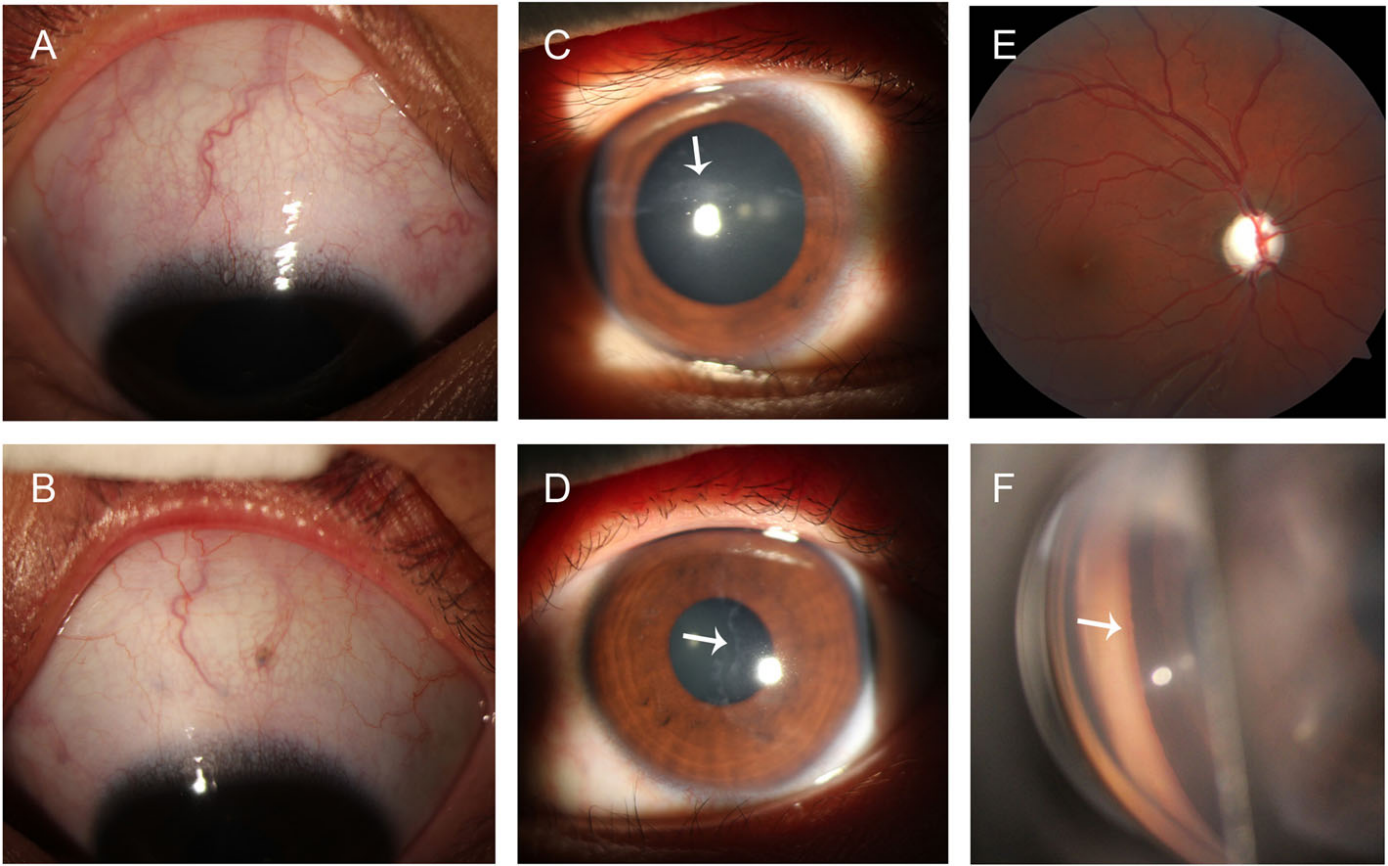
Ophthalmic findings. Slit-lamp photography of the patient’s (**a** & **c**) right eye and (**b** & **d**) left eye showing (**a** & **b**) conjunctival and episcleral vessels, (**c** & **d**) corneal Haab’s striae (arrows) and (**c**) dilated pupil. **e** Fundus examination of the right eye showing torturous retinal vessels and vertical cup disc ratio of 1.0. **f** Gonioscopy examination of the left eye showing an open anterior chamber angle with blood in Schlemm’s canal (arrow)

Ophthalmic examinations showed a Snellen visual acuity of 20/2000 in the right eye (OD) and 20/125 in the left eye (OS). The best-corrected visual acuity of OD had improved to 20/200 (− 5.50 × 90°) and OS to 20/100 (− 1.50–1.00 × 95°). Intraocular pressure (IOP) was 58 and 42 mmHg for OD and OS, respectively. Slit-lamp examination revealed torturous and dilated conjunctival and episcleral vessels, Haab’s striae, enlarged corneal diameter (13.5 mm) bilaterally and a relative afferent pupillary defect in OD (Fig. 3a-d). Bilateral torturous retinal vessels and vertical cup to disc ratio of 1.0 were found (Fig. 3e). Gonioscopy showed an open angle with blood in Schlemm’s canal bilaterally (Fig. 3f). The subfoveal choroidal thickness of OD was 627 μm and OS was 683 μm as measured by optical coherence tomography. The axial length of OD was 23.95 mm and OS was 23.40 mm, and the central corneal thickness of OD was 597 μm and OS was 598 μm.

Notwithstanding the negative result of neurological examination, we made a diagnosis of SWS based on the cutaneous finding and ocular involvement.

After a three-week outpatient drug treatment with maximum IOP-lowering medications (fixed combination of latanoprost 0.005% and timolol 0.5% once a day, brinzolamide 1% twice a day and brimonidine 0.2% twice a day in both eyes), trabeculectomy with intraoperative application of mitomycin C was performed in both eyes due to the uncontrolled IOP (OD: 39 mmHg and OS: 23 mmHg). No adverse event was found postoperatively, except for the tendency of subconjunctival fibrosis of the bleb and elevated IOP (24-40 mmHg) in both eyes. Subconjunctival injections of 5-fluorouracil 5 mg were given weekly within the early postoperative follow-up (in total, 4 injections in OD and 5 injections in OS). Over next 3-month follow-up period, the IOP was controlled ranging from 12 to 16 mmHg bilaterally, and no IOP-lowering drugs were needed.

## Discussion and conclusions

SWS is a rare sporadic disease affecting multiple organs. Although capillary malformation (CM) has been suggested participating in the syndromic alterations, the pathogenesis remains elusive. Recent studies have confirmed the guanine nucleotide-binding protein G(q) subunit alpha (*GNAQ*) R183Q mutation in 90% of SWS patients [10], which possibly causes CM via increasing endothelial cell proliferation [11]. Somatic *GNAQ* mutation is enriched in endothelial cells of CM isolated from skin, brain and choroid. How these cells would be affected by the mutation is yet to be known. Other postulations include the persistence of primordial sinusoidal vascular channels and the altered innervation of perivascular vessels due to the neural crest cell abnormalities [12].

Clinically, the nevus flammeus shows various phenotypes, most frequently affecting the region innervated by one or more branches of the fifth cranial nerve unilaterally, with an extracraniofacial distribution only in 29% of cases [2, 13]. However, Waelchli R [11] suggested that the lesions appeared to follow the embryonic vascular placode, rather than the dermatome. The embryological theory suggested that the time and site of the mutation determine the bilaterality and the regions of the lesions, which, to some extent, accounts for the co-occurrence of forehead PWS and neurological and ocular abnormalities. Vascular abnormalities of the conjunctiva, episclera, retina and choroid (“tomato catsup” fundus) are more commonly observed in an eye with PWB involving the upper eyelid. Exudative retinal detachment and macular edema can be secondary to the diffuse choroidal hemangioma. SWS-associated glaucoma is likely to be the result of the developmental anomaly of the anterior chamber angle and/or the elevated episcleral venous pressure [1].

The neurological symptoms of SWS are believed to be the results of vascular stasis and poor perfusion in the cortex beneath the leptomeningeal CM [3, 10]. Furthermore, the disruption of hypothalamic-pituitary axis by SWS could also lead to endocrine complications, including growth hormone deficiency [9] and hypothyroidism [7]. However, the reported muscular involvement in SWS, such as idiopathic inflammatory myopathy [5] and rhabdomyolysis [8], should be more aligned with the term “co-morbidity”, rather than “complication”. It is believed that both muscles and vessels develop from the same germ layer (mesoderm) and could be suffered from the effect of *GNAQ* mutation simultaneously. Greene et al. found that frequency of overgrowth was high in the patients with SWS, including diffuse soft-tissue thickening associated with CM [13]. For skeletal involvement, the maxillo-facial osteohypertrophy could be secondary to the intra-osseous vascular malformation or localized growth factor production [13]. A cause-and-effect relationship was suggested between the overlying cutaneous PWS and the localized skeletal hypertrophy [6, 13]. Besides, the syndactyly, believed as the consequence of disturbance in interdigital tissue apoptosis [4], could be associated with the *GNAQ* mutation, which leads to cell proliferation and inhibition of apoptosis in SWS.

In this case, the patient showed ocular abnormalities and cutaneous nevus flammeus, and was diagnosed as SWS. No evident endocranial involvement was found. In addition to the vision-threatening glaucoma, attention should be paid to the extensive PWS that distributed not only in bilateral faces, but also in left neck and left upper limb. Notably, the limb PWS were located on the radial side of the forearm, adjacent to the incomplete duplication of thumb anatomically. SWS co-existing with polydactyly has not been previously described, and their relationship remains elusive. Nevertheless, we propose to explain this possible phenomenon based on the following evidences. First, the number of digital rays in the limbs during the morphogenesis of the digits depends on the amount of tissue cells available, and the source of the limb mesoderm is composed by the somatopleura, the migration of muscle precursors and the progressive invasion by endothelial and nerve cells [14]. Capillary endothelial cell proliferation and malformation in SWS could play a role in tissue enriching, shown as soft-tissue and skeletal overgrowth [13], which could contribute to the polydactyly development. Second, the number of digits is determined by the Sonic hedgehog (*SHH*) gene, which is mediated by the Gli family of Zn-finger transcription factors [14]. The *SHH* signaling could be accumulated in the tissues with CM, caused by *GNAQ* mutation, due to vascular stasis and altered function of exchange and transport [10], which results in excess duplication of digits.

Limitations of the case report were as follow. Firstly, the co-occurrence, even though catching much attention, can be just an accidental event since there was just a single case. Secondly, without genetic testing, there was a lack of identifiable evidence and the presumption above has yet to be confirmed.

In summary, this report presented a case with unrecognized co-existence of SWS and polydactyly. Abnormal proliferation of capillary endothelial cell in SWS could be related to duplication of thumb, especially in the case with PWS involving the adjacent skin. However, the etiology of this co-occurrence is unclear. Therefore, further investigation is required to explore the potential pathogenesis.

## Data Availability

All data analyzed or generated during the study are included in this published article.

## Abbreviations

SWS: Sturge-Weber syndrome
PWS: Port-wine stains
OD: Right eye
OS: Left eye
IOP: Intraocular pressure
CM: Capillary malformation
GNAQ: Guanine nucleotide-binding protein G(q) subunit alpha
SHH: Sonic hedgehog
